# A six stage operational framework for individualising injury risk management in sport

**DOI:** 10.1186/s40621-017-0123-x

**Published:** 2017-09-20

**Authors:** Mark Roe, Shane Malone, Catherine Blake, Kieran Collins, Conor Gissane, Fionn Büttner, John C. Murphy, Eamonn Delahunt

**Affiliations:** 1 School of Public Health, Physiotherapy and Sports Science, University College Dublin, Dublin 4, Ireland; 2 Gaelic Sports Research Centre, Department of Science, Institute of Technology Tallaght, Dublin, Ireland; 3 School of Sport, Health and Applied Science, St Mary’s University, London, UK; 4 Medfit Proactive Healthcare, Dublin, Ireland; 5 Institute for Sport and Health, University College Dublin, Dublin, Ireland

**Keywords:** Injury risk management, Operational framework, Etiology model, Injury prevention, Athletic performance, Athlete management

## Abstract

Managing injury risk is important for maximising athlete availability and performance. Although athletes are inherently predisposed to musculoskeletal injuries by participating in sports, etiology models have illustrated how susceptibility is influenced by repeat interactions between the athlete (i.e. intrinsic factors) and environmental stimuli (i.e. extrinsic factors). Such models also reveal that the likelihood of an injury emerging across time is related to the interconnectedness of multiple factors cumulating in a pattern of either positive (i.e. increased fitness) or negative adaptation (i.e. injury).

The process of repeatedly exposing athletes to workloads in order to promote positive adaptations whilst minimising injury risk can be difficult to manage. Etiology models have highlighted that preventing injuries in sport, as opposed to reducing injury risk, is likely impossible given our inability to appreciate the interactions of the factors at play. Given these uncertainties, practitioners need to be able to design, deliver, and monitor risk management strategies that ensure a low susceptibility to injury is maintained during pursuits to enhance performance. The current article discusses previous etiology and injury prevention models before proposing a new operational framework.

## Background

Managing injury risk is important for maximising athlete availability and performance. Although athletes are inherently predisposed to musculoskeletal injuries by participating in sports, etiology models have illustrated how susceptibility is influenced by repeat interactions between the athlete (i.e. intrinsic factors) and environmental stimuli (i.e. extrinsic factors) (Meeuwisse 1994; Meeuwisse et al. 2007). Such models also reveal that the likelihood of an injury emerging across time is related to the interconnectedness of multiple factors cumulating in a pattern of either positive (i.e. increased fitness) or negative adaptation (i.e. injury) (Bittencourt et al. n.d.; Windt and Gabbett 2016).

The process of repeatedly exposing athletes to workloads in order to promote positive adaptations whilst minimising injury risk can be difficult to manage. Etiology models have highlighted that preventing injuries in sport, as opposed to reducing injury risk, is likely impossible given our inability to appreciate the interactions of the factors at play. Thus, practitioners must accept some degree of uncertainty despite their best efforts to minimise injury risk (Windt and Gabbett 2016). Given these uncertainties, practitioners need to be able to design, deliver, and monitor risk management strategies that ensure a low susceptibility to injury is maintained during pursuits to enhance performance. The current article discusses previous etiology and injury prevention models before proposing a new operational framework.

## Etiology models

In 1994 Meeuwisse proposed a linear, causal pathway to illustrate the onset of injury. This involved a predisposed athlete characterised by intrinsic factors (e.g. age, previous history, neuromuscular control level) becoming susceptible to injury via interactions with extrinsic risk factors (e.g. game conditions, playing equipment) (Meeuwisse 1994). It was proposed that these risk factors would influence the athlete’s tolerance to inciting events and to the mechanism attributable to the onset of injury.

Meeuwisse et al. later recognised that a linear approach containing a start and an end point does not reflect the true onset of injury in sport and proposed a recursive cycle where repeated participation occurs in the absence of injury (Meeuwisse et al. 2007). This revised model more accurately reflected the frequent exposures to activities associated with sporting seasons whilst illustrating that the same factors and mechanisms may have different outcomes (i.e. injury or continued participation) for different athletes.

Bittencourt et al. expanded upon the dynamic nature of injury risk in a conceptual framework to counteract the reductionist approach of simplifying the many factors surrounding the onset of injury into separate units (i.e. biomechanical, behavioral, physiological and psychological) (Bittencourt et al. n.d.). It was proposed that units of varying magnitudes of influence interact and collectively create a “web of determinants”. In turn, this would influence the athlete’s response to their environment leading to the emergence of an injury or positive adaptation. The recursive elements of these models highlight the influence of positive (e.g. increased aerobic capacity) and negative (e.g. injury) responses on an athlete’s ever-evolving injury risk in sports. However, despite physiological systems underpinning many areas of human performance, and injury healing, Bittencourt was the first to place great emphasis on supercompensation (i.e. positive physiological changes associated with exposures to stressful stimuli and recovery).

Simultaneously, Windt and Gabbet expanded on the Bannister fitness-fatigue model proposed as previous etiology models failed to adequately account for the workloads associated with training and competition (Windt and Gabbett 2016). Indeed, the workload—injury aetiology model illustrated a paradox that workloads can both decrease injury risk by increasing fitness, or increase injury risk by inducing fatigue or maladaptations. It was proposed that the careful application of appropriate workload and recovery were required to manage injury risk and optimise supercompensation.

## Injury risk management models

Although these models help illustrate the elements influencing the onset of injury they do not, by design, promote the development of injury risk management strategies. In 1987 van Mechelen outlined a four-stage sequence for preventing injuries. These included establishing the extent of the problem using epidemiology data (step 1), establishing the cause and mechanism of injury (step 2), introducing preventative measures (step 3), and assessing intervention efficacy by repeating step 1 (step 4) (van Mechelen et al. 1992).

Finch later added additional steps to assist in the translation of research into injury prevention practise (TRIPP model) to include a description of the intervention context to inform implementation strategies (step 5) and evaluation of the intervention via ‘real-world’, as opposed to solely scientific analytics (step 6) (Finch 2006).

## From etiology and prevention models to an operational framework

Interventions derived from current etiology models tend to be group-based such as standardised warm-ups or rule changes. Although some group-based initiatives have been shown to be effective it is possible that injury risk management may be enhanced with personalised interventions (Al Attar et al. 2016). For instance, diversity within a team squad means each athlete presents with unique characteristics which modifies their susceptibility to injury. Hence, implementing a generic injury prevention protocol, or clustering athletes into groups based on the presence or absence of certain variables, may not reduce injury risk if certain factors unique to each athlete are not addressed.

**Fig. 1:**
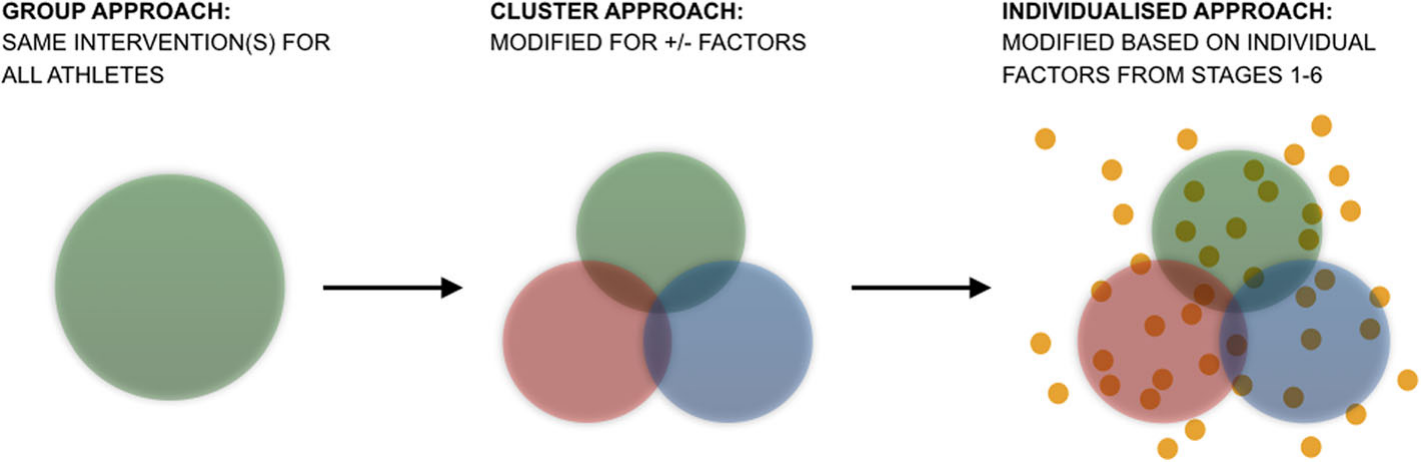
Strategic approaches to injury risk management

**Fig. 2:**
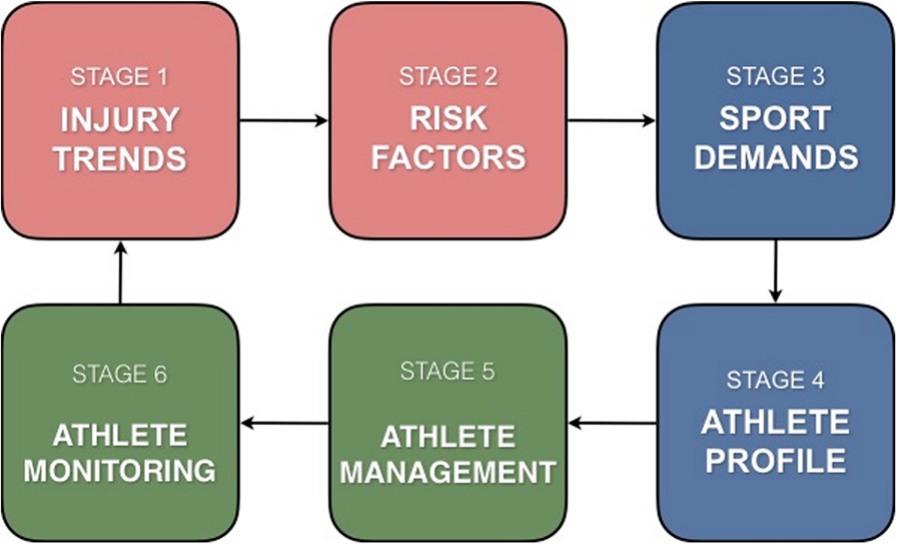
Proposed operational framework for managing injury risk. Legend: The six stage operational framework outlines how awareness of injury trends and risk factors (stage 1 and 2), profiling the demands of a sport and the capabilities of the athlete (stage 3 and 4), and monitoring the athlete’s responses to evidence-based interventions (stage 5 and 6) can guide practitioners in managing injury risk

An operational framework to guide practitioners in continuously managing injury risk whilst considering factors unique to the athlete’s sport and profile has yet to be proposed in a manner facilitating supercompensation. Thus, the current article builds on previous etiology and prevention models to propose a novel paradigm (Fig. 1). The six stage operational framework outlines how awareness of injury trends and risk factors (stage 1 and 2), profiling the demands of a sport and the capabilities of the athlete (stage 3 and 4), and monitoring the athlete’s responses to evidence-based interventions (stage 5 and 6) can guide practitioners in managing injury risk. The authors propose that this novel framework can build on the success of group based interventions (Fig. 2).

## Stage 1 – injury trends: when, where, and how do certain athletes sustain certain injury?

**Fig. 3:**
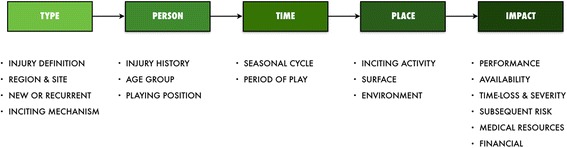
Data for understanding when, where, and how do certain athletes sustain certain injury

Stakeholders need to understand the incidence (e.g. rate per 1000 exposure hours, number of injuries per athlete each season) and prevalence (proportion of population affected) of common medical attention and time-loss injuries across different stages of the season. Such information can ensure that resources are targeted towards common injuries whilst promoting compliance with specific risk management strategies. Thus, the initial step involves awareness of when, where, and how certain athletes sustain specific injuries in the sport (Fig. 3).

Accounting for differences relating to the onset of injury (e.g. seasonal cycle, inciting activity, inciting mechanism, probability of injury within defined time-periods) between age-groups, playing position, and previously injured athletes may assist in this process. Such information will assist in identifying injury patterns and athletes at increased risk. Understanding the impact of injury on athlete availability, perceived performance, and probability of sustaining a future injury before risk management strategies are embedded into team programmes will ensure that realistic expectations exist among stakeholders.

## Stage 2 – injury risk factors: what factors heighten or migitate injury risk?

Whilst stage one focuses on synthesising information related to the onset of common injuries in a sport, stage two identifies factors influencing the likelihood that an injury will be sustained. This involves practitioners seeking information on factors that alter risk of incurring specific injuries, whilst appreciating that the interactions between certain variables can modify the impact of individual risk factors. For instance, acute spikes in training load increase injury risk, however, greater aerobic fitness decreases susceptibility (Malone et al. 2016). Similarly, previous injury increases risk of incurring a future injury, however, confounding variables such as neural inhibition, selective muscle atrophy, alterations in fascicle length, strength deficits, and increased susceptibility to fatigue need to be considered. Such maladaptations may exist in some athletes following rehabilition periods which may alter their injury susceptibiity following return to play. Thus, practitioners should avoid reliance on a single risk factor when managing injury risk.

Indeed, coupling factors (e.g. physiological, musculoskeletal, competition schedules) related to injury suggests that a multidisciplinary approach is required to indentify and assess the relevant non-modifiable or modifiable factors at play (Bittencourt et al. n.d.; Windt and Gabbett 2016). Such approaches may increase the likelihood that athletes are provided with opportunities to complete beneficial training, receive adequate recovery, and participate in competition in a manner not compromising their welfare or injury risk. Whilst this stage may not directly involve intervention, it identifies key factors to consider in latter stages that influence an athlete’s risk of sustaining a musculoskeletal injury.

## Stage 3 – sport demands: what does the athlete need to be prepared for?

Understanding the demands of each training and competition cycle over consecutive seasons provides clarity on which tasks athletes are to be prepared for. The measurement of work rate (e.g. GPS), physiological responses (e.g. heart rate), and subjective markers of load (e.g. sRPE) may be useful. Such information provides insight into the physiological characteristics required to sustain peak performance including time-dependent peak work-rate (e.g. m/s, m.min^−1^), as well as loads experienced across both acute (≤ 7 days) and more chronic (≥8 days) phases (Windt and Gabbett 2016). Individualised measures will allow practitioners establish personalised graded exposures to elicit supercompensation. Whilst appreciation of these demands does not ensure accurate risk assessment stage 3 promotes an understanding of the demands placed on athletes during different activities and stages of the season. This can assist practitioners in reducing the risk of an athlete being inappropriately exposed to demands they are ill-prepared for, thereby decreasing maladaptation (i.e. fatigue and injury) (Windt and Gabbett 2016).

## Stage 4 – athlete profile: does the athlete present with characteristics of at risk and/or successful athletes?

After identifying injury risk factors, a series of assessments can be undertaken to investigate whether an individual presents with the characteristics athletes more susceptible to injuries identified in stage 1. Assessing whether the athlete possesses the desired physical characteristics associated with participating at a given level of the sport may also reflect preparedness and identify opportunities to increase performance. This aspect is particularly important for managing return-to-play or early career athletes transitioning to a more elite playing standard to reduce the likelihood deficits may impair the performance.

It should be noted that there tends to be overlap in characteristics between athletes that do, and do not, sustain injury (Bahr 2016). However, this stage identifies factors unique to the athlete’s profile that may need addressing to reduce injury risk and maximise their availability across consecutive seasons.

## Stage 5 – athlete management: what are favourable short and long-term interventions?

Stage 5 combines information gathered in previous stages with the practitioners experiences and knowledge of efficacious scientific literature, to identify suitable interventions for developing components of an athlete’s profile. With such information practitioners are best positioned to address a key question: “how do we develop the required physical characteristics to prepare the athlete for the demands of their sport without increasing injury risk?”

Interventions have been identified to reduce risk of new and recurrent injuries, as well as to develop related factors such as neuromuscular control, force, and aerobic capacity (Al Attar et al. 2016; Buchheit and Laursen n.d.). When interventions have been identified, practitioners can justify a progressive series of activities that load the relevant physiological systems to promote supercompensation and increase performance of desired tasks. Awareness of responses to training or match-play demands, including recovery time-lines, will be essential for planning subsequent activities.

## Stage 6 – athlete monitoring: how does the athlete respond overtime?

The aim of stage 6 is to understand how the athlete responds to stage 5 in order to manipulate future interventions. For instance, monitoring changes in injury risk and performance within defined time-periods associated with an intervention can help manage upcoming cycles. Such information can assist practitioners in coordinating other aspects of the athletes programme to maximise supercompensation whilst making sure efficacious interventions to reduce injury risk have been embedded into performance-orientated programmes.

Incorporating reliable and sensitive measures in stage 6 provides important information to objectively evaluate the effectiveness of specific interventions. Basic statistics may assist practitioners in calculating and interpreting dose-response relationships such as incidence, odds ratio, relative risk, probability, correlations, meaningful changes, and magnitude of effect size. Efforts should also be undertaken to aggregate league-wide, multi-year data to yield larger sample sizes for analysing the impact of multiple variables on injury risk. In the meantime, practitioners can combine monitoring of injury data (step 1), sporting demands (step 3), athlete profiling (step 4), and intervention outcomes (step 6) to guide future risk management strategies for the athletes in their care.

## Conclusions

The operational framework may be useful in managing injury risk throughout the sporting season by considering each athlete’s characteristics before designing appropriate interventions. Examples of variables to consider at stages 1-6 are outlined in Table 1. This article does not intend to identify every related variable, nor does it imply that eradication of injuries is possible if stages 1-6 are followed. Rather it proposes an operational framework to guide practitioners in applying an evidence-based approach to injury risk management by decreasing the likelihood of particular injuries occuring because risk factors were addressed according to an athlete characteristics and sporting demands. Although the unpredictable nature of certain elements of sport requires consideration throughout the injury risk management process, stakeholders must ask themselves “are we doing all we can to reduce injury risk while maintaining optimal performance for the athlete and team?”

**Table 1:** Example of variables to consider in stages 1-6 when managing the injury risk of an elite midfield Gaelic football player. The player is aged 21 years and sustained a hamstring injury within the last 12 months

Stage 1 – Injury Trends	Stage 2 – Risk Factors	Stage 3 – Sport Demands	Stage 4 – Athlete Profiling	Stage 5 – Athlete Management	Stage 6 – Athlete Monitoring
Injury probability > 12 months uninjured, 14% Aged 21-24, 27% Previous injury, 41% Previous hamstring injury, 44%	Injury history Previous injury, Odds risk, OR = 3.0 Previous hamstring injury, OR = 3.3	Match-play demands Total distance, 10,621m Relative total distance, 152 m.min^−1^ High speed distance (>17 km/h), 2000 m High speed distance (>17 km/h), 29 m.min^−1^	Physiological characteristics Incremental treadmill test YoYoIR1 YoYoIR2 30-15 IFT 1 km time trial Repeated-sprint fatigue index Knee flexor strength Biomechanical assessment Anthropometric assessment	Interventions Nordic hamstring exercise programme Small-sided game variations High-intensity interval training Load management iTRIMP thresholds	Useful tools Load management GPS running distances Force-velocity profile iTRIMP thresholds Knee flexor strength S-RPE Acute:chronic workload ratio HAGOS Wellness Heart rate recovery
Team rate Time-loss injuries per season, 42 Players affected per season, 27 Injury prevalence per season, 66%	Training variables > 15 max velocity exposures, OR = 3.4 > 120m max speed running, OR = 3.1 Acute:chronic workload ratio > 1.35, OR = 2.0 Acute:chronic workload ratio > 2.0, OR = 3.4 Weekly S-RPE >2700 AU, OR = 2.4	Match-play worse case scenario Relative total distance in 1 min, 252 m.min^−1^ Relative total distance in 5 min, 197 m.min^−1^			
Injury incidence Training, 4/1000 h Match-play, 62/1000 h Match-play to training rate ratio, 15.3	Physiological variables 1 km time trial time > 3.1 min, OR = 2.5 YoYoIR1 distance 1670-2150 m, OR = 1.6 YoYoIR1 distance > 2150m, OR = 0.33 > 20 mins at 85%HR_max_ per week, OR = 0.22 Preseason eccentric hamstring strength between-limb imbalance > 10%, RR = 1.4	Training demands Pitch session, 370 S-RPE AU Gym session, 250 S-RPE AU Small-sided game for 4 min, 930m			
Common injuries Lower limb, 76% (mean, 28 time-loss days) Hamstring, 21% (mean, 26 time-loss days)					
Common injury mechanism Non-contact, 81%					
Average associated time-loss Per injury, 28 days Per injury for 21-24 years, 24 days Per team each season, 908 days Injury burden, 115 days/1000 h					
Injuries Per Cycle Preseason, 17% Competitive cycle 1, 25% Mid-season, 19% Competitive cycle 2, 39%					
